# An Immobilized Rh‐Based Solid Molecular Catalyst for the Reductive Hydroformylation of 1‐Octene

**DOI:** 10.1002/anie.202424144

**Published:** 2025-06-04

**Authors:** Keanu V. A. Birkelbach, Jeroen T. Vossen, Thorsten Rösler, Isabella Kappel, Ansgar Meise, Marc Heggen, Andreas J. Vorholt, Regina Palkovits

**Affiliations:** ^1^ Institute for Technical and Macromolecular Chemistry RWTH Aachen University Worringerweg 2 52074 Aachen Germany; ^2^ Institute for a Sustainable Hydrogen Economy Forschungszentrum Jülich Marie‐Curie‐Str. 5 52428 Jülich Germany; ^3^ Max Planck Institute for Chemical Energy Conversion Stiftstraße 34–36 45470 Mülheim an der Ruhr Germany; ^4^ Max‐Planck‐Institut für Kohlenforschung Kaiser‐Wilhelm‐Platz 1 45470 Mülheim an der Ruhr Germany; ^5^ Ernst Ruska‐Centre for Microscopy and Spectroscopy with Electrons (ER‐C) Forschungszentrum Jülich GmbH 52428 Jülich Germany

**Keywords:** Catalyst recycling, Heterogeneous catalysis, Homogeneous catalysis, Polymers, Reductive hydroformylation, Solid molecular catalysts

## Abstract

The reductive hydroformylation of olefins is an important process in the chemical industry to produce alcohols directly without isolating the aldehydes as intermediates. As the hydroformylation is a homogeneously catalyzed reaction, the catalyst recycling and down‐stream processing is often complex and energy intensive. A heterogeneous reductive hydroformylation catalyst was developed in this work by immobilizing Rh on polymeric amine macroligands to form solid molecular catalysts (SMCs). An iterative macroligand improvement was carried out by increasing the basicity and number of amine groups at the immobilization sites. With the best performing SMC, olefins were fully converted to >99% alcohols without a hydrogenation of the substrate in a solvent free environment, thus requiring only a separation of the heterogeneous catalyst to yield the pure product. The catalyst was successfully recycled over 12 runs with a perpetual Rh leaching as low as 1.2%, and the metal to macroligand ratio was identified as most important parameter in reducing metal loss.

## Graphical abstract

The reductive hydroformylation of olefins can be catalyzed by Rh which is immobilized on a highly branched amine containing polymer for efficient recycling and a high selectivity and alcohol yield.

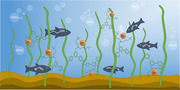

## Introduction

**Scheme 1:**
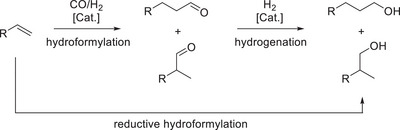
Hydroformylation, hydrogenation, and concerted reductive hydroformylation reactions.

In terms of production volume, the hydroformylation is currently one of the largest homogeneously catalyzed industrial processes. State‐of‐the‐art hydroformylation catalysts are commonly based on Rh or Co and feature carbonyl and/or phosphine‐derived ligands.^[^ ^1^ ^]^ The reaction entails the 100% atom‐efficient addition of synthesis gas, a mixture of CO and H_2_, to an olefin to yield the corresponding C_+1_ aldehydes (Scheme 1). These aldehydes represent a versatile chemical platform which enables subsequent transformations to carboxylic acids via oxidation, to amines by reductive amination or to alcohols via hydrogenation, to name a few. These consecutive synthetic steps can be combined with the hydroformylation in a one‐pot tandem reaction system, allowing for a reduction in investment and operating costs.^[^ ^2^, ^3^ ^]^ For the combination of hydroformylation and hydrogenation in the conversion of olefins to alcohols, this process is referred to as reductive hydroformylation.

Early approaches toward the reductive hydroformylation involved the use of two functionally different catalysts, one for each of the individual reaction steps. These can facilitate their respective transformations under one set of reaction conditions, such as a Rh and Ru‐based tandem catalyst systems,^[^ ^4^, ^5^ ^]^ or each transformation under differing conditions, such as the sulfoxanthphos‐Rh SILP/silica‐supported Shvo's catalyst by Bell et al.^[^ ^6^ ^]^ or the modified Rh(PPh_3_)/Raney‐Ni tandem by Masdeu‐Bultó et al.^[^ ^7^ ^]^ Homogeneous auto‐tandem catalysts which facilitate both partial reactions have also been reported.^[^ ^8^, ^9^ ^]^ Amongst the most active are Co‐based catalysts,^[^ ^10^, ^11^, ^12^ ^]^ or Rh‐based systems with appropriate phosphine or tertiary amine ligands. The latter were used by Vorholt et al., showing the influence of the amine concentration, its basicity, and cone angle on the reductive hydroformylation activity and selectivity.^[^ ^13^ ^]^ To recover the catalyst after the reaction, various multiphase approaches showed promising results, either as liquid/liquid multiphase reaction systems,^[^ ^14^, ^15^ ^]^ or through an immobilization of the Rh catalyst in a supported ionic liquid phase.^[^ ^16^ ^]^ Besides these, the catalyst can also be separated *post reactio*, for example, by changing the phase behavior through the injection of CO_2_ to form a second polar phase.^[^ ^17^ ^]^ Despite these considerable advances, catalyst leaching and the high boiling point of the products remain a challenge in multiphase reaction systems when separating the products and recovering the metal catalyst. The use of a heterogeneous catalyst would address such leaching and separation issues. However, the hydroformylation and thus reductive hydroformylation are only catalyzed homogeneously, with few examples of the successful immobilization of such catalysts.^[^ ^18^, ^19^, ^20^ ^]^ A novel approach is offered by so‐called solid molecular catalysts (SMCs), which aim to combine the advantages of hetero‐ and homogeneous catalysis.^[^ ^21^, ^22^, ^23^ ^]^ They are commonly comprised of a polymer backbone containing defined ligand sites, i.e., a macroligand, which coordinates a molecular metal precursor. This yields a heterogeneous material with a molecularly defined active site. Herein, an SMC based on an amine‐containing polymer used to immobilize a Rh catalyst is presented for heterogeneously catalyzed reductive hydroformylation reactions. A variation in polymer design allows for an iterative improvement of the polymer macroligand by modulating the environment of the metal center. After an optimization of the reaction parameters, a highly active and selective catalyst was obtained and its recyclability was shown in batch reactions.

## Results and Discussion

Based on previous findings by Vorholt et al. regarding the Rh/amine catalyst system in the reductive hydroformylation,^[^ ^13^ ^]^ amine moieties were implemented in a polymer backbone which can support the active Rh species. The synthesis of the polymers was investigated first, and their reactivity in the reductive hydroformylation was assessed afterwards.

### Polymer Design and Synthesis

**Figure 1:**
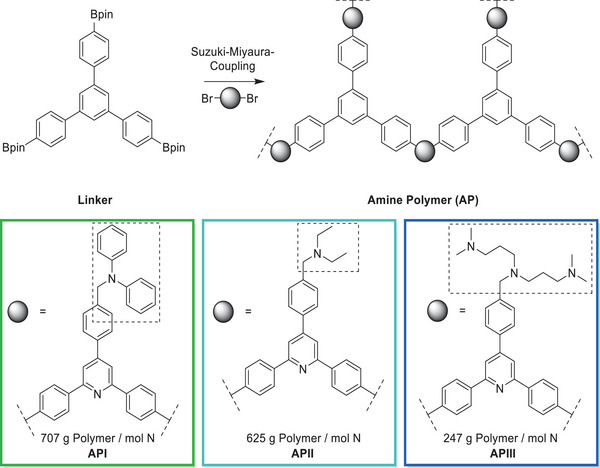
Monomers and synthetic approach toward amine polymers (**AP**). Also shown is the mass of polymer per mole of non‐pyridinic nitrogen, with the marked areas highlighting the N considered for each polymer. For **APIII**, the tertiary amine structure was synthesized after polymerization.

**Scheme 2:**
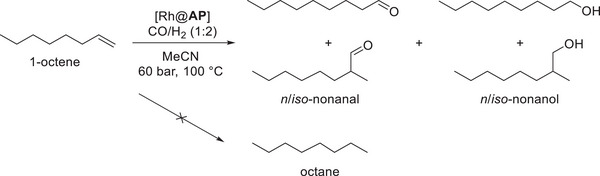
Screening of Rh@**AP** in the reductive hydroformylation of 1‐octene (0.23 g, 2.0 mmol, 5 wt% mesitylene as internal standard IS)) in MeCN (2 mL), in situ catalyst formation with [Rh(acac)(CO)_2_] (0.5 mol% referred to the substrate, Rh:N = 1:3 for **API** and **APII**, Rh:N = 1:9 for **APIII**), 100 °C, 60 bar (CO:H_2_ = 20:40), 700 rpm, 24 h, in 20 mL autoclave reactors. Analyzed by GC FID and XRF.

**Figure 2:**
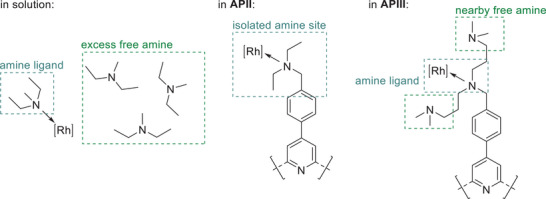
Proposed environments of the active metal complex in solution (left) and in SMCs based on **APII** (middle) and **APIII** (right). Coordinating amine ligands are highlighted in teal, free amine is highlighted in green.

Three polymers (**API** to **APIII**) with tertiary amines were synthesized to serve as macroligands in SMCs (see Figure 1). In the case of **APII**, the respective monomer containing the tertiary amine was synthesized via an amended trimerization route and polymerized to yield the final macroligand.^[^ ^24^ ^]^ For **API** and **APIII**, a tolyl‐containing trimer was synthesized, polymerized, and later functionalized to yield the desired macroligands. The polymers showed no significant porosity, as determined by N_2_ physisorption, and reactions are assumed to take place on the polymer surface. From these polymers and the metal precursor [Rh(acac)(CO)_2_], the catalysts Rh@**AP** were formed in situ in the reductive hydroformylation. Initial conditions were chosen based on those established by Vorholt et al. with acetonitrile as a solvent (Scheme 2).^[^ ^13^ ^]^ When employing the catalyst Rh@**API**, no reductive hydroformylation activity toward the alcohols was observed and an aldehyde yield of 93% with an *n*‐aldehyde selectivity of 37% was obtained (0.5 mol% Rh, Rh:N of 1:3, 60 bar CO:H_2_ (1:2), 100 °C, 24 h reaction time in acetonitrile). Based on the available knowledge of the homogeneous system, the aromatic methyl diphenylamine sites in **API** are expected to be unsuitable as ligands: they are not basic enough (their basicity is comparable to the pyridine ring within the polymer structure p*K*_a_ ≈  5.23),^[^ ^13^ ^]^ and their cone angle is too large. To address both issues, the phenyl groups of **API** were replaced with sterically less demanding ethyl groups in **APII**, resulting in an amine site with a p*K*_a_ of ca. 10.35. When employing Rh@**APII**, the reductive hydroformylation of 1‐octene was observed, albeit with a low alcohol yield of 6% and an aldehyde yield of 90% under the same reaction conditions as **API** over 24 h. Based on the homogeneous system, **APII** should offer an ideal amine ligand both in terms of basicity and cone angle. However, the amine concentration was also found to have a major effect on the hydroformylation activity of the homogeneous analogue, with the highest activity and selectivity toward alcohols obtained in almost pure amine as a solvent.^[^ ^13^ ^]^ To emulate this, **APIII** was synthesized with the goal of increasing the amine density around the active site of the polymer from one to three amine functionalities (Figure 2). When employing Rh@**APIII**, the yield was increased to 20% alcohols and 60% aldehydes in only 4 h reaction time (Rh:N changed from 1:3 to 1:9 due to the additional amine groups in close proximity). In 24 h reaction time, an alcohol yield of 81% and an aldehyde yield of 19% were obtained and the 1‐octene was fully converted. With no significant differences in the basicity of the individual sites between **APII** and **APIII**, the number of amine moieties is the major distinction and thus the main driving force of the reductive hydroformylation activity.^[^ ^25^ ^]^ As shown in previous studies by Vorholt et al., the amine sites partake in the catalysis as ligand and additionally as a counterion and/or hydrogen source.^[^ ^13^ ^]^ The Rh precursor in the presence of synthesis gas can catalyze the hydroformylation in solution, but does not show any hydrogenation activity, as shown in blank experiments (Table S1). This result suggests that the hydroformylation can take place with the immobilized and dissolved Rh, while the hydrogenation can only be catalyzed by the immobilized species. The forced amine proximity in **APIII** enables this behavior, and Rh@**APIII** was chosen as catalyst in all reactions moving forward.

### Reductive Hydroformylation

**Figure 3:**
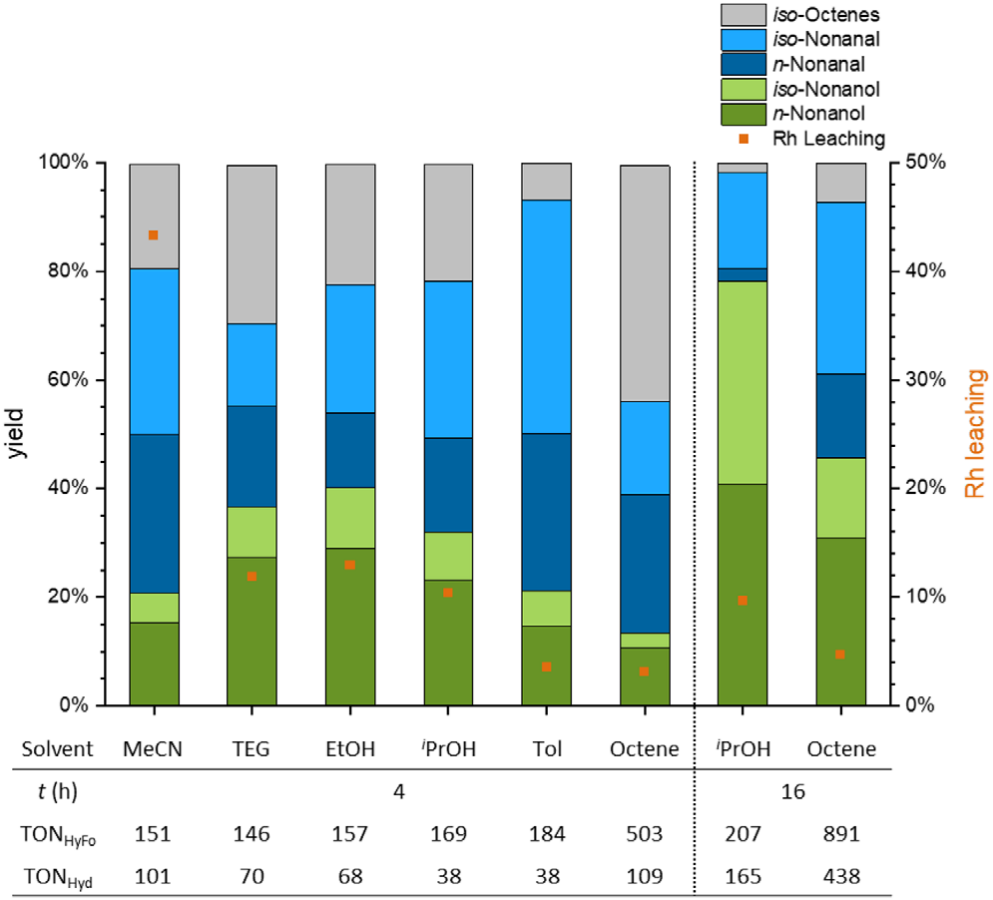
Yield, Rh leaching and TONs in the solvent variation in the reductive hydroformylation of 1‐octene (0.33 mL, 5 wt% mesitylene as internal standard (IS)), 2 mL solvent, in situ catalyst formation with **APIII** (247 g mol_N_^−1^) and [Rh(acac)(CO)_2_] (0.5 mol% referred to the substrate, Rh:N = 1:9), 100 °C, 60 bar (CO:H_2_ = 20:40), 700 rpm, 4 h, in 20 mL autoclave reactors. Analyzed by GC FID.

Acetonitrile did not prove to be an ideal solvent as evidenced by a Rh leaching of 44% in the reaction employing Rh@**APIII**. This may be due to the ability of acetonitrile to act as a ligand in metal complexes. Additionally, MeCN provides a high hydroformylation but only a low hydrogenation activity. Thus, a solvent variation was carried out. Polar protic solvents such as triethyleneglycol (TEG), EtOH and *iso*‐propanol are known to be beneficial in hydrogenation reactions^[^ ^26^ ^]^ and showed an alcohol yield around 35% and aldehyde yield around 50%, significantly higher compared to acetonitrile (Figure 3). The Rh leaching was also reduced significantly to ca. 10% for each of the solvents. However, the handling and work‐up of the catalyst proved difficult in TEG due to its high viscosity and a swelling of the polymer. The non‐polar aprotic solvent toluene was tested, which showed the highest hydroformylation yield (70%) but only provided a hydrogenation yield similar to acetonitrile (20%). The leaching in toluene was reduced even further to only 4%. As the reaction does not require a polar protic solvent but also works in non‐polar solvents such as toluene with a lower catalyst leaching, the reaction was also tested in neat conditions in 1‐octene. This resulted in a decrease in catalyst concentration relative to the substrate amount from 0.5 mol% with solvents to 0.1 mol% Rh. Conclusively, while 1‐octene does not perform best in terms of yield with ca. 10% alcohols and 45% aldehydes compared to other solvents, it still exhibited a much higher TON in the hydroformylation (TON_HyFo_) of 503 and in the hydrogenation (TON_Hyd_) of 109 in comparison to the systems employing an additional solvent such as *iso*‐propanol with 157 (TON_HyFo_) and 68 (TON_Hyd_). Neat conditions amount to a multitude of process‐related advantages, particularly in regard to product workup. Hence, neat conditions were used in all future experiments.

**Figure 4:**
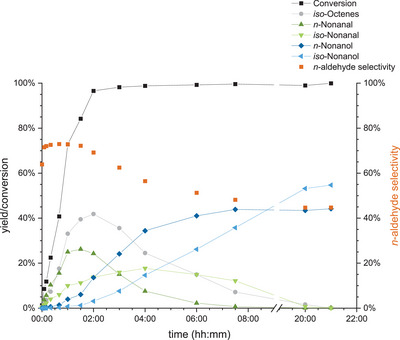
Yield and conversion in the time profile in the reductive hydroformylation of 1‐octene (118 mL, 5 wt% mesitylene (IS)) with an in situ catalyst formation with amine polymer III (247 g mol_N_^−1^) and [Rh(acac)(CO)_2_] (0.1 mol% referred to the substrate, Rh:N = 1:9) at a pressure of 60–100 bar (CO:H_2_ = 1:2) and 800 rpm stirring in a 300 mL reactor. Analyzed by GC FID.

**Figure 5:**
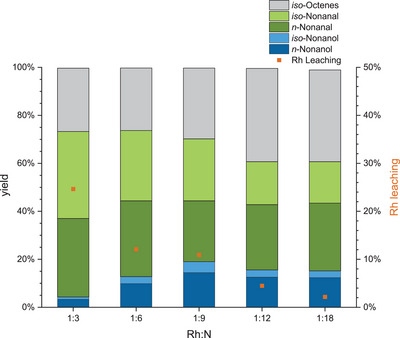
Yield and Rh leaching of the rhodium to amine variation in the reductive hydroformylation of 1‐octene (2 mL, 5 wt% mesitylene (IS)) with an in situ catalyst formation with amine polymer III (247 g mol_N_^−1^) and [Rh(acac)(CO)_2_] (0.1 mol% referred to the substrate, Rh:N = variable) at a pressure of 75 bar (CO:H_2_ = 25:50), 100 °C and 700 rpm stirring speed for 4 h in 20 mL autoclave reactors. Analyzed by GC FID and X‐ray fluorescence spectroscopy (XRF).

**Figure 6:**
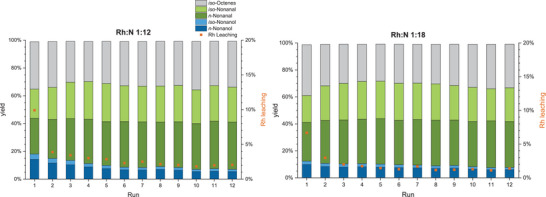
Yield and Rh leaching of the catalyst recycling (left: Rh:N ratio of 1:12, right: Rh:N ratio of 1:18) in the reductive hydroformylation of 1‐octene (2 mL, 5 wt% mesitylene (IS)) with an in situ catalyst formation with amine polymer III (247 g mol_N_^−1^) and [Rh(acac)(CO)_2_] (0.1 mol% referred to the substrate) at a pressure of 75 bar (CO:H_2_ = 25:50) and 700 rpm stirring speed for 4 h in 20 mL autoclave reactors. Analyzed by GC FID and XRF.

Blank experiments employing only the macroligand **APIII** did not show any conversion at all (Table S1). After an additional variation of reaction pressure and gas composition and a temperature variation were carried out (Figure S1 and S2), a time‐profile reaction was carried out in a larger 300 mL reactor on a 120 mL scale to investigate the kinetics of the reaction. Within 2 h reaction time, 1‐octene is fully converted to the aldehydes and *iso*‐octenes (Figure 4). The conversion to *n*‐nonanal was much faster than to *iso*‐nonanal due to its higher reactivity. The highest *n*:*iso* ratio was reached within 1.5 h of the reaction with 73:27. As all 1‐octene was converted, the remaining olefins were mostly converted to *iso*‐aldehydes, resulting in a decrease in *n*:*iso* ratio to 45:55 at the end of the reaction. In parallel, the hydrogenation of *n*‐nonanal to *n*‐nonanol also commenced faster than for *iso*‐nonanal. If the slower hydrogenation of the *iso*‐aldehydes was a concentration effect due to the slower hydroformylation or a steric effect is unclear. After 20 h, full conversion with >99% alcohol yield can be obtained. This again shows that the conversion in neat substrate at a large scale is successful and superior over diluted systems. It also shows that the *n*:*iso* ratio observed during batch experiments is not constant and depends on the progress of the reaction. Regarding the recycling of the immobilized Rh catalyst, different metal to amine ratios were tested by varying the amount of **APIII** added to the reaction. For the variation, the mass of polymer per mole of non‐pyridinic nitrogen was considered for the Rh:N ratio (ref. Figure 1). The highest Rh loading of Rh:N of 1:3 investigated in this study corresponds to one Rh atom for every single trifunctional amine functionalization in the polymer. As shown in Figure 5, the hydrogenation activity of Rh@**APIII** increases from a Rh:N of 1:3 ratio to 1:9 and remains comparable up to 1:18. Rh leaching decreases significantly at higher macroligand amounts, from 25% at a 1:3 ratio to 2% for a 1:18 ratio. This correlates with a lower share of dissolved Rh in favor of a coordinated species. To further elucidate the influence of the metal to polymer ratio, batch recycling experiments encompassing 12 runs were performed at Rh:N ratios of 1:12 and 1:18 (Figure 6). In both cases, the SMCs maintained catalytic activity throughout all 12 runs. At the lower ratio of 1:12, the first run of the recycling exhibited the highest hydroformylation and hydrogenation activity, but also the highest Rh leaching of 10%. In the subsequent runs, the hydrogenation activity of the system sees a slowing decline, while the hydroformylation activity remains largely constant. The Rh leaching decreases significantly after the first run and stabilizes at around 2% per run after the fifth reaction. Until the conclusion of the recycling, the Rh leaching stays consistently at that level within the margin of error. In the recycling experiment with a Rh:N ratio of 1:18, the initial leaching is significantly lower at 7% and stabilizes at around 1.3% in the fourth run onwards. This highlights the importance of the amount of polymer as a variable reaction parameter to reduce leaching. In total, the experiments amounted to a TON_HyFo_ of 7722, TON_Hyd_ of 1184 and a total Rh leaching of 38% with a Rh:N ratio of 1:12 and a total TON_HyFo_ of 8150, TON_Hyd_ of 1142 and Rh leaching of 24% with a Rh:N ratio of 1:18.

### Analysis of Used Catalyst

**Figure 7:**
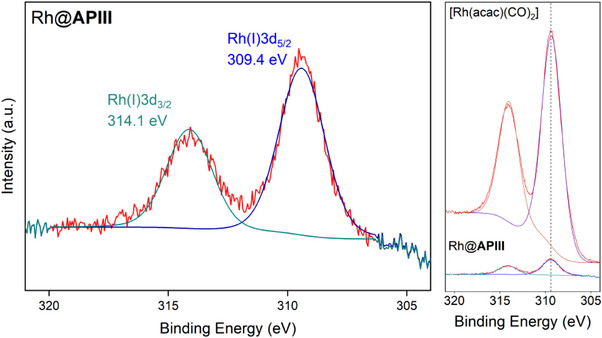
XPS Rh3d high‐resolution core‐level spectrum of used Rh@APIII (left) and a comparison between used Rh@**APIII** (right, bottom) and [Rh(acac)(CO)_2_] (right, top).

**Figure 8:**
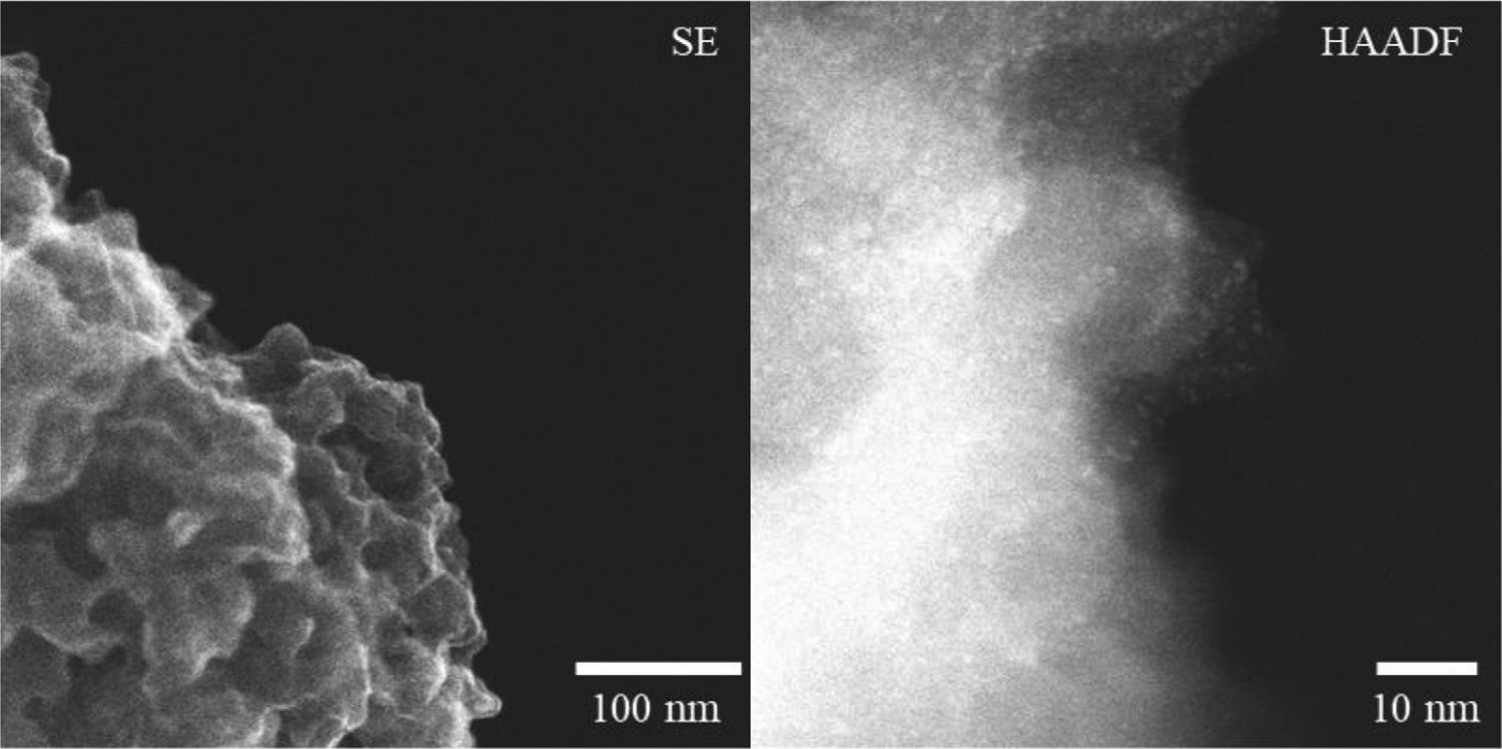
AC‐SE (left) and AC‐HAADF‐STEM (right) image of used Rh@**APIII** showing no visible large‐scale metal agglomerates (left) and finely dispersed Rh of two different sizes on higher magnification (right).

**Figure 9:**
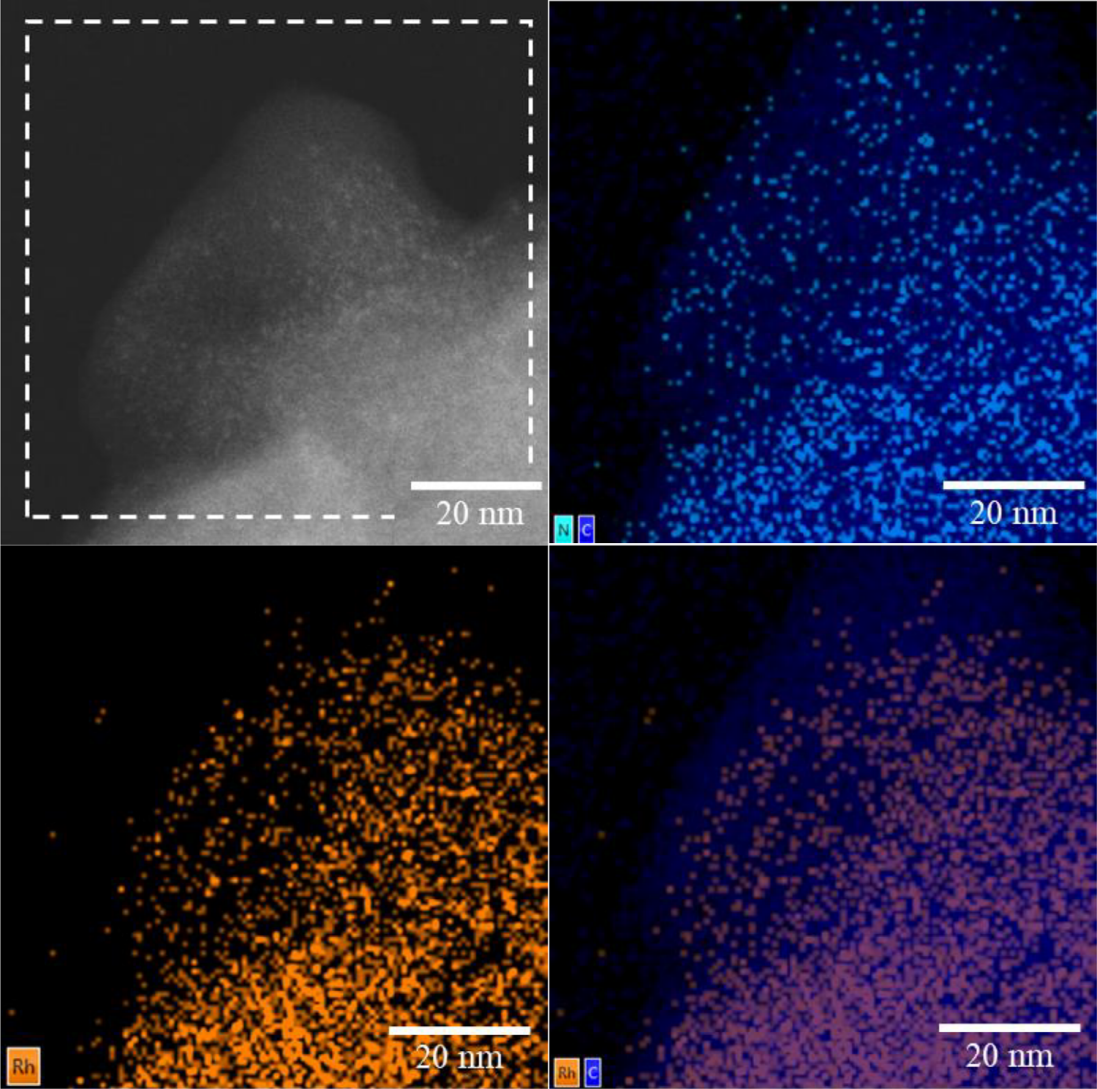
HAADF‐STEM (top left) and ‐EDX (rest) images of used Rh@**APIII** showing the polymer being composed of a carbon backbone with nitrogen functionalization (top right) and Rh finely dispersed across the polymer surface with some areas of small‐scale agglomeration (bottom).

The used SMC Rh@**APIII** was obtained from the previously shown, upscaled batch reaction. X‐ray photoelectron spectroscopy (XPS) Rh3d high‐resolution spectra revealed that Rh is contained in the used catalyst in oxidation state + 1 (Figure 7). In addition, a comparison between the metal precursor and the used catalyst shows good alignment of the two Rh3d signals of both samples, evident of a similar electronic environment for both. To further substantiate this, aberration‐corrected (AC‐) high‐angle annular dark field‐ (HAADF‐) scanning transmission electron microscopy (STEM) and ‐energy dispersive X‐ray (‐EDX) analysis was done on the used catalyst samples. Figure 8 shows used Rh@**APIII** at two levels of magnification. At lower magnification (Figure 8, left, secondary electron mode (SE) a spherical particle with a rugged surface is revealed. Importantly, no large‐scale metal agglomerates are visible on the particle surface, which is consistent with the findings of XPS analysis. At higher magnification (Figure 8, right), bright spots representing metal sites of two different sizes are revealed. The first, smaller species, can be attributed to Rh single sites. The exact nature of the single site cannot be differentiated based on the microscopy images. Based on its size, the second species can be attributed to small multinuclear agglomerates. Importantly, no lattice structures are visible, excluding the possibility of metallic agglomerates. Instead, a likely explanation is multinuclear rhodium/carbonyl complexes. The co‐existence of both single atoms and such multinuclear complexes has been previously observed with the homogeneous system,^[^ ^13^ ^]^ and mobility of the coordinated metal species across the polymer surface was observed with related SMCs.^[^ ^27^ ^]^ HAADF‐STEM‐EDX was used to further analyze the surface composition of the used catalyst and the dispersion of the metal species (Figure 9). The underlying electron image again shows the co‐existence of Rh sites of different sizes (Figure 9, top left). In the center bottom of the image, the topography of the polymer particle likely adds some depth effects to the EDX analysis. As C (dark blue) makes up the backbone of the macroligand with repeating N (light blue) functionalization, the EDX mapping of N and C represents a good match (Figure 9, top right). For Rh, a fine dispersion can be seen (Figure 9, bottom left), which mostly coincides with the dispersion of N on the polymer. Superimposing the occurrence of Rh with that of carbon (Figure 9, bottom right) reveals that the Rh is finely dispersed across the entirety of the polymer particle.

**Figure 10:**
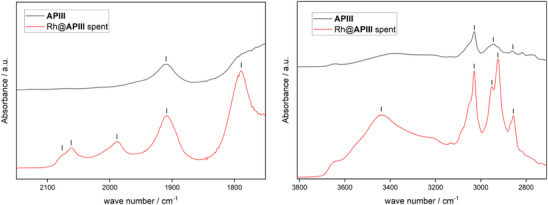
Stacked excerpts of DRIFTS‐IR measurements of macroligand **APIII** (grey) and the used SMC Rh@**APIII** (red) focusing on the carbonyl vibrational stretch area (left) and the N─H and O─H vibrational stretch areas (right).

Lastly, diffuse reflectance infrared Fourier transform spectroscopy‐ (DRIFTS‐) infrared (IR) spectra were recorded to investigate the presence of Rh carbonyl species via their characteristic CO stretching vibrations between 1800–2100 cm^−1^ (Figure 10). In said area, the pristine polymer **APIII** shows a singular defined peak at 1909 cm^−1^. Since **APIII** does not contain CO, this signal is likely caused by a functionality in the polymer. In contrast, the respective spectrum of Rh@**APIII** after the reaction features five signals, evidencing the uptake of carbonyl compounds during the reaction. Qualitative observations made for the corresponding homogeneous system were used as basis for their assignment.^[^ ^13^ ^]^ As shown in Figure 10, the first three signals are found as a shoulder at 2076, 2061, and 1988 cm^−1^ which can be attributed to uncharged rhodium complexes and are assumed to be formed initially upon coordination of the precursor by the macroligand. A signal at 1909 cm^−1^ was found in both samples, but is significantly more pronounced in the used catalyst. It also displays asymmetry in Rh@**APIII**, suggesting an overlap of two species: one of the macroligand and one of a carbonyl species at slightly lower wave numbers. The lower wavelength signal matches the anionic complex [Rh(CO)_4_]^−^, the formation of which was previously observed during the analogous homogeneous reaction and assigned a species at 1904 cm^−1^.^[^ ^28^ ^]^ An additional signal at 1790 cm^−1^ may be caused by the formation of multinuclear, anionic rhodium carbonyl complexes which include bridging CO ligands.^[^ ^13^, ^29^ ^]^ The formation of such multinuclear anionic Rh complexes aligns with the results obtained from XPS, high‐resolution imaging and now DRIFTS‐IR. The formation of these anionic Rh complexes would require counterions, such as the protonated amine functionalization of **APIII**. To verify their presence, the O─H and N─H stretching vibration areas of the macroligand and used catalyst were also compared via DRIFTS‐IR. For the pristine polymer, three signals can be seen between the wave numbers of 3030 and 2858 cm^−1^ (Figure 10, right), which can be assigned to a partial protonation of the amine groups in the macroligand. This protonation took place during the workup after the synthesis and was not fully removable during a subsequent stirring in base due to limited site availability.^[^ ^30^ ^]^ In comparison, in the Rh@**APIII** sample these signals are significantly more pronounced, and two additional signals can be seen. While the signals below 3000 cm^−1^ are partially caused by product adsorbed on the polymer, the signal at around 3050 cm^−1^ is not. As such, this points toward an increased protonation during the reaction. As no obvious source of protonation was present during the reductive hydroformylation, their formation as counterions represents the best explanation for the observed changes. An additional broad peak between 3600 and 3200 cm^−1^ can again be assigned to the O─H stretching vibration of nonanol. As for the acac ligand of the metal precursor, two signals in the area between 1500 and 1600 cm^−1^ were expected.^[^ ^31^ ^]^ These signals were not found in Rh@**APIII** (Figure S4). The absence of these signals proves the abstraction of the acac ligand from the metal center during the coordination by the macroligand in the reductive hydroformylation.

## Conclusion

In this work, a solid molecular catalyst for the reductive hydroformylation was presented. Rh was immobilized on an amine‐based polymer functioning as a macroligand for the Rh catalyst. Iterative improvements were carried out on the structure of the polymer to increase the basicity from **API** to **APII** and the number of proximal amine groups from 1 to 3 in **APIII**. Rh@**APIII** was shown to be the best‐performing catalyst with the highest reductive hydroformylation activity in subsequent reactions. The reaction could be conducted in neat substrate without the need for additional solvents, reaching full conversion of 1‐octene to the alcohols. This enabled a simple catalyst recycling after the reaction via centrifuging without the need for further product purifications as full conversion of the olefins to alcohols is achieved. The Rh:N ratio played an essential role to reduce the Rh leaching. In recycling experiments, Rh@**APIII** maintained its reactivity over 12 recycling runs and showed a low perpetual Rh leach of just over 1%. The nature of the immobilized Rh catalyst was investigated using a combination of XPS, HAADF‐STEM(‐EDX), and IR‐DRIFTS analysis. High resolution microscopy revealed the co‐existence of finely dispersed Rh sites of two different sizes across the polymer surface. IR‐DRIFTS comparisons between the macroligand **APIII** and the used SMC revealed the abstraction of the acac ligand during the reaction and confirmed the presence of carbonyl species in the used catalyst. Importantly, the analysis of the carbonyl region suggested the co‐existence of single sites and mono‐ and multi‐nuclear, neutral, or anionic Rh/CO complexes.

In future investigations, the polymer can be varied further to improve the steric demand, basicity, and potentially influence the *n:iso* selectivity. Additionally, the role of the amine in the polymer as a ligand and counter ion can be investigated in detail. Besides batch recycling reactions, a continuous recycling set up can be developed to investigate the long‐term leaching and behavior of the catalyst and the reaction.

## Supporting Information

The supporting information containing additional graphics, detailed experimental procedures and analytical data is available free of charge (PDF).

## Conflict of Interests

The authors declare no conflict of interest.

## Supporting information



Supporting Information

## Data Availability

The data that support the findings of this study are available from the corresponding author upon reasonable request.
