# Supplementary material for: An Immobilized Rh‐Based Solid Molecular Catalyst for the Reductive Hydroformylation of 1‐Octene
Source: Angew Chem Int Ed Engl. 2025 Jun 4;64(33):e202424144. doi: 10.1002/anie.202424144 (PMC12338383; doi:10.1002/anie.202424144)
Supplement: Supplementary file 1 — Supporting Information [file ANIE-64-e202424144-s001.pdf]

An Immobilized Rh-based Solid Molecular Catalyst for the Reductive Hydroformylation of 1-Octene

Keanu V. A. Birkelbach,[a,b]* *Jeroen T. Vossen,*[a,c,]* *Thorsten Rösler,*[a,c] *Isabella Kappel,*[b,d] *Ansgar Meise,*[e] *Marc Heggen,*[e] *Andreas J. Vorholt,*[c,@] *Regina Palkovits*[a,b,c,@]

[a] Institute for Technical and Macromolecular Chemistry, RWTH Aachen University, Worringerweg 2, 52074 Aachen, Germany.

[b] Institute for a Sustainable Hydrogen Economy, Forschungszentrum Jülich, Marie-Curie-Str. 5, 52428 Jülich, Germany

[c] Max Planck Institute for Chemical Energy Conversion, Stiftstraße 34–36, 45470 Mülheim an der Ruhr, Germany.

[d] Max-Planck-Institut für Kohlenforschung, Kaiser-Wilhelm-Platz 1, 45470 Mülheim an der Ruhr, Germany

[e] Ernst Ruska-Centre for Microscopy and Spectroscopy with Electrons (ER-C), Forschungszentrum Jülich GmbH, 52428 Jülich, Germany

* Both authors contributed equally to this work.

@ andreas-j.vorholt@cec.mpg.de, palkovits@itmc.rwth-aachen.de

Containing:

1 Table

15 Figures

18 Pages

Contents

1 Supporting Data and Graphs 2

2 Experimental 6

3 GC FID Chromatograms 17

4 References 18

1 Supporting Data and Graphs

Table S1: Conversion and yields in percent in blank experiments employing only [Rh(acac)(CO)$_2$] or **APIII**.

	X(1-Oct)	X(Oct)	Y(*i*-Oct)	Y(Octane)	Y(*n*-Ald)	Y(*i*-Ald)	Y(*n*-Alc)	Y(*i*-Alc)
Precursor	99.97	96.42	3.55	0	42.72	53.39	0.11	0.21
**APIII**	0	0	0	0	0	0	0	0

Conditions: Precursor or **APIII**, 100 °C, 75 bar pressure (CO:H_2 25:50), 700 rpm, 4 h, neat.

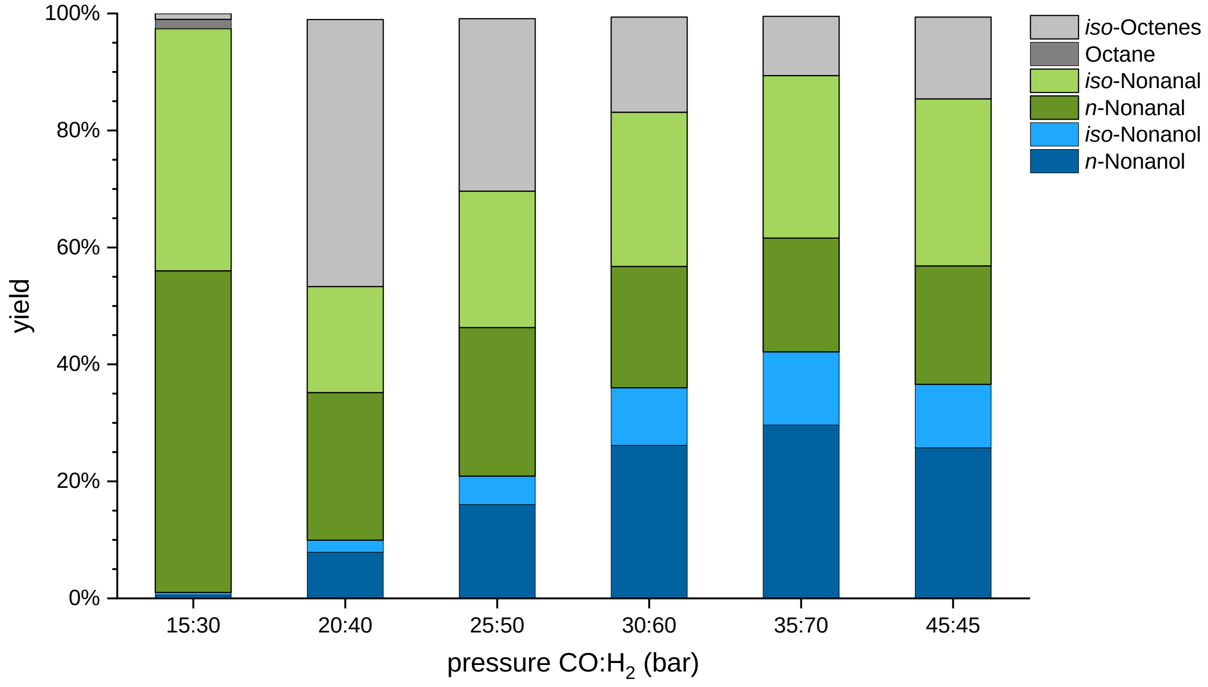

Figure S1: Yield in the pressure variation in the reductive hydroformylation of 1-octene (2 mL, 5 w% mesitylene as internal standard (IS)) with an *in situ* catalyst formation with **APIII** (247 g mol_N^{-1}) and [Rh(acac)(CO)$_2$] (0.1 mol% referred to the substrate, Rh:N = 1:9) at 100 °C and 700 rpm stirring speed for 4 h in 20 mL autoclave reactors. Analyzed by GC FID.

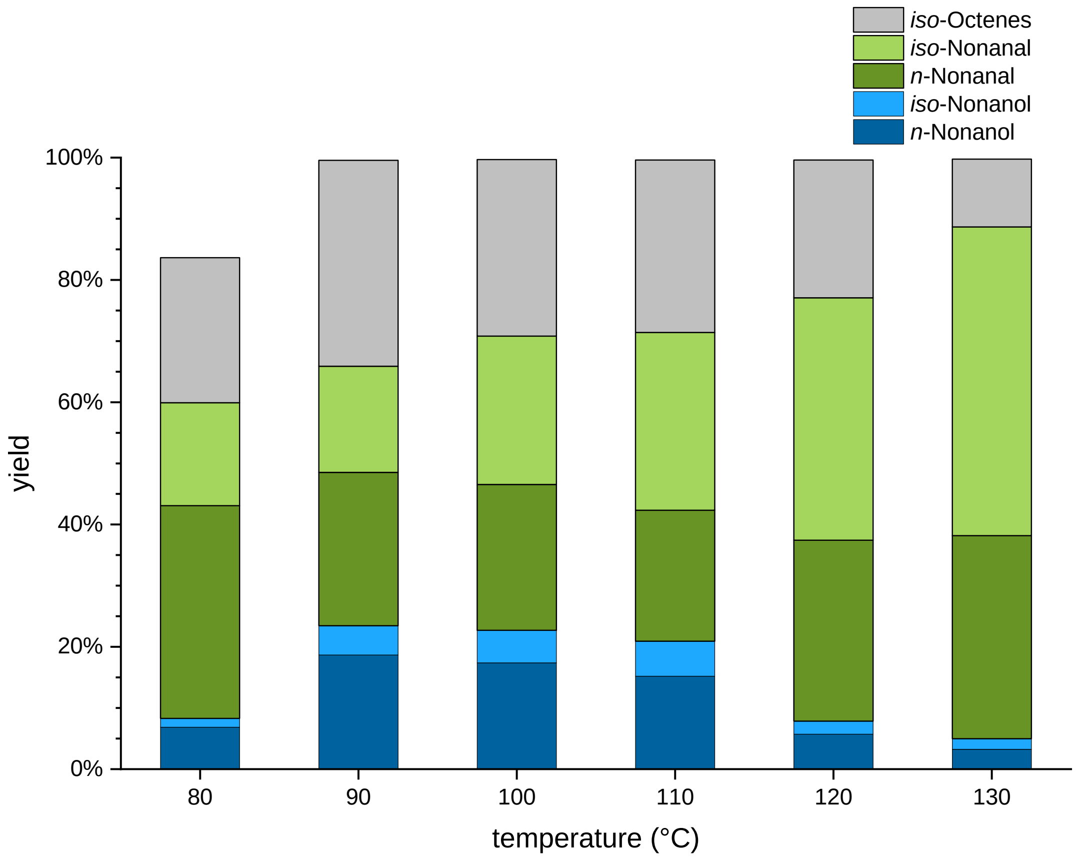

Figure S2: Yield in the temperature variation in the reductive hydroformylation of 1-octene (2 mL, 5 w% mesitylene (IS)) with an *in situ* catalyst formation with **APIII** (247 g mol_N^{-1}) and $[Rh(acac)(CO)_2]$ (0.1 mol% referred to the substrate, Rh:N = 1:9) at a pressure of 75 bar ($CO:H_2$ = 25:50) and 700 rpm stirring speed for 4 h in 20 mL autoclave reactors. Analyzed by GC FID.

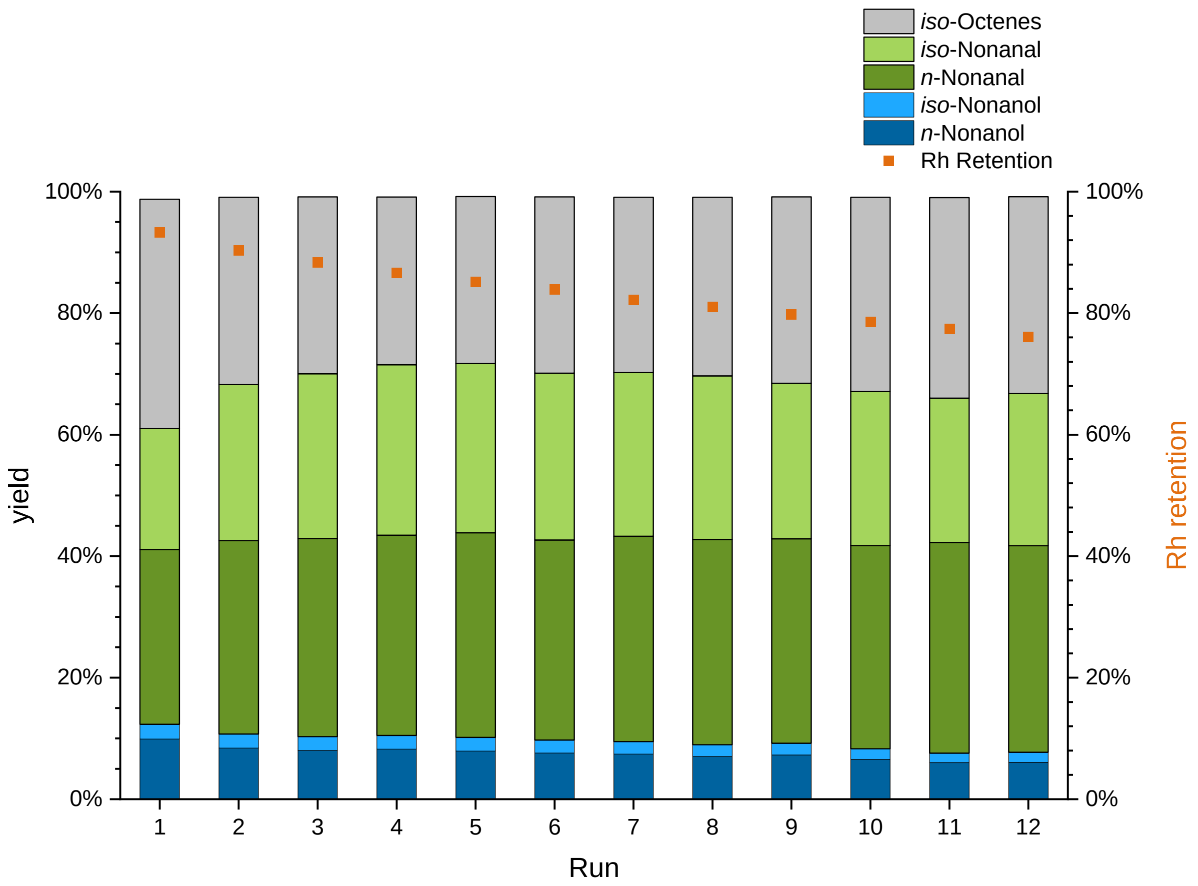

Figure S3: Yield and Rh retention of the catalyst recycling in the reductive hydroformylation of 1-octene (2 mL, 5 w% mesitylene (IS)) with an *in situ* catalyst formation with **APIII** (247 g mol_N^{-1}) and $[Rh(acac)(CO)_2]$ (0.1 mol% referred to the substrate, Rh:N = 1:18) at a pressure of 75 bar ($CO:H_2$ = 25:50) and 700 rpm stirring speed for 4 h in 20 mL autoclave reactors. Analyzed by GC FID and XRF.

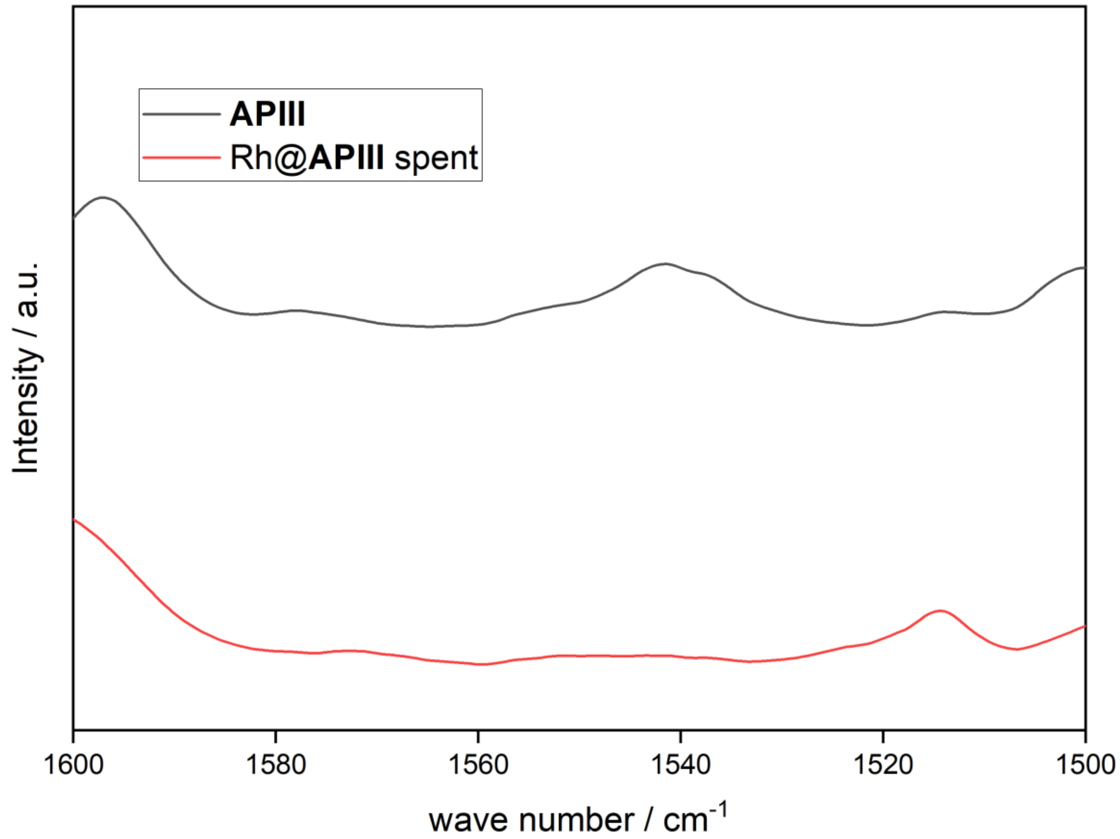

Figure S4: Stacked excerpts of DRIFTS-IR measurements of macroligand **APIII** (grey) and the used SMC Rh@**APIII** (red) focusing on the fingerprint area of the acac ligand.

2 Experimental

General considerations

The polymer synthesis and analysis were carried out at the RWTH Aachen, while the reductive hydroformylation reactions were conducted at the Max Planck Institute for Chemical Energy Conversion.

For the synthesis of the macroligands tolylaldehyde, KOH, NaOH, $Pd(PPh_3)_4$, bromobenzene, phenylboronic acid pinacol ester, *N*,*N*-diisopropyl ethylamine and diphenyl amine were purchased from *Sigma Aldrich*/ *Merck*. 4-Bromoacetophenone was purchased from *TCI*. Aqueous ammonia solution (25%), HCl and hydrogen peroxide solution (30%) were purchased from *Labsolute*. Diethylamine was purchased from *Roth*. Caesium carbonate was purchased from *fluoro chem*. NBS was purchased from *Glentham Life Sciences*. AIBN was purchased from *Fluka Anal.*. All chemicals were used as obtained from the vendors.

For the catalytic experiments, mesitylene was obtained from *Alfa Aesar* and the solvents were purchased from *Carl Roth*. All other chemicals were obtained from *Sigma Aldrich*. All chemicals were used as obtained from the vendors and degassed before application in the catalytic reactions. Carbon monoxide (99.997% purity) and hydrogen (99.999% purity) for catalytic reactions were obtained from *AirLiquide*.

GC measurements of the catalysis experiments were conducted on a *Nexis GC-2030* purchased from *Shimadzu* with a flame ionization detector. A *GCMS-QP2020* by *Shimadzu* was used to conduct GC with in-line mass spectrometry measurements. For both apparatus *Rtx-1* columns of the company *Restek* with a length of 30 m, an inner diameter of 0.25 mm and a particle size of 0.5 µm were installed and helium was used as a carrier gas. The Sternberg method was applied to approximate GC factors for non-calibrated substances.

XRF measurements were conducted on a *Xepos C* by the company *Spectro*. Samples were placed in the detector in containers with a Prolene 12 µM foil in between. Measurements were carried out for 600 seconds in a He/air atmosphere with an X-ray energy level of 3 keV to 19 keV. A Co-Pd-alloy X-ray source and a silicon-drift-detector were used. Samples were analyzed for rhodium content.

For TEM sample preparation, the powder specimen was suspended and dispersed in Milli-Q Water and deposited on a holey carbon Cu TEM grid applying the drop-cast method. The suspension was evaporated under ambient lab conditions. Residual hydrocarbon contamination was removed using a UV-based sample cleaner (*Hitachi HT ZONETEM II*). In order to distinguish the active species and the morphology of the present catalysts, aberration corrected

scanning transmission electron microscopy (*AC-STEM*) was conducted on a *Hitachi HF5000* and a *FEI TITAN ChemiSTEM*, both, operating at 200 kV and equipped with a spherical-aberration (Cs) probe corrector. Energy dispersive x-ray spectroscopy (EDX) was performed on a *ThermoFisher Super-X EDX* system.

IR spectra of the used catalyst were collected using a *Bruker VERTEX 70* spectrometer with a *Harrick* praying mantis DRIFTS cell at atmospheric pressure and ambient temperature.

N_2 physisorption was measured using a *Quadrasorb SI* unit from *3P Instruments* at -196 °C. Prior to the measurement, the samples were degassed for 6 h in a *FloVac* degasser at 60 °C. The data was evaluated by means of the *QuadraWin* software. For all macroligands, little porosity was determined, and in case of **APII** and **APIII** the recorded values were on the extreme low end of the device specifications. In any case, no trend between porosity and catalytic activity was observable. Rather than report these values, the macroligands are referred to as non-porous and all reactions are assumed to take place on the polymer surface.

Thermogravimetric analysis (TGA) was carried out on a *STA 6000-TGA* measuring device from *Perkin Elmer*. The measurements were performed in a temperature range from 30 °C to 850 °C at a heating rate of 10 °C/min. A N_2 -flowrate of 150 mL/min was used during measurement.

General Procedure for the Synthesis of Monomers

Trimerization reactions were carried out using a literature procedure.[1]

API and **APIII**: 4-Tolylaldehyde (688 mg, 5,73 mmol, 1 eq) and 4-bromoacetophenone (2,51 g, 12,62 mmol, 2,2 eq) were dissolved in 50 ml EtOH. To this solution, KOH (845 mg) was added. After 10 min of stirring, the solution had turned dark red, and 20 ml of 25 % aqueous NH_3 solution was added. The solution was stirred for 3 days, during which a heavy precipitation was formed. The precipitate was filtered off and washed with cold EtOH:H_2O 1:1 until the washing solution remained colorless. The product was received as a pale powder (1,24 g, 45,3 % yield).

^{1}H NMR (300 MHz, $CDCl_3$) δ 8.11 – 8.00 (m, 4H), 7.86 (s, 2H), 7.64 (m, *J* = 8.3, 1.5 Hz, 6H), 7.35 (d, *J* = 7.8 Hz, 2H), 2.45 (s, 3H).

^{13}C NMR (75 MHz, $CDCl_3$) δ 156.44, 150.94, 139.73, 138.02, 135.63, 132.04, 130.10, 128.93, 127.16, 123.94, 117.28, 21.45.

APII: 4-(Bromomethyl) benzaldehyde (2 g, 10 mmol, 1 eq) and diethyl amine (2,43 g, 30 mmol, 3 eq) were dissolved in 50 ml THF and refluxed for 2 h. Afterwards, the solvent was removed and the received residue partitioned between diethyl ether and H_2O. The organic phase was then extracted with 1M HCl, and the resulting aquatic phase neutralized with 2M NaOH. The product was extracted with 2×100 ml diethyl ether and washed with saturated NaCl. The product was received as clear oil after distillation (1,73 g, 90,6 % yield). NMR was in agreement with literature.[2]

4-((diethylamino)methyl)benzaldehyde (0,956 g, 5 mmol, 1 eq) and 4-bromoacetophenone (3,00 g, 15,00 mmol, 3,0 eq) were dissolved in 50 ml EtOH. To this solution, KOH (1000 mg) was added. After 10 min of stirring, the solution had turned dark red, and 20 ml of 25 % aqueous NH_3 solution was added. The solution was stirred for 3 days, during which a heavy precipitation was formed. The precipitate was filtered off and washed with cold EtOH:H_2O 1:1 until the washing solution remained colorless. The product was received as a pale-yellow powder (1,13 g, 41,2 % yield).

^{1}H NMR (300 MHz, $CDCl_3$) δ 8.06 (d, *J* = 8.7 Hz, 4H), 7.87 (s, 2H), 7.66 (m, *J* = 10.9, 8.4 Hz, 6H), 7.51 (d, *J* = 8.3 Hz, 2H), 3.65 (s, 2H), 2.57 (q, *J* = 7.1 Hz, 4H), 1.08 (t, *J* = 7.2 Hz, 6H)

^{13}C NMR (75 MHz, $CDCl_3$) δ 156.54, 150.64, 141.77, 138.43, 137.13, 132.02, 129.76, 128.80, 127.07, 123.75, 117.18, 57.39, 46.99, 11.93.

General Procedure for the Synthesis of Polymers

The appropriate monomer (1 eq), 1,3,5-tris(4-pinacolatoborolanephenyl)benzene (0.67 eq), caesium carbonate (1.5 eq respective to bpin) and $Pd(PPh_3)_4$ (0.05 eq) were dissolved in degassed DMF with 1% water under inert gas atmosphere. The reaction mixture was stirred at 80 °C for 24h. Then, bromobenzene (0.5 eq) was added to endcap the polymer and stirred at 80 °C for an additional 24h. This was repeated with phenylboronic acid pinacol ester (1.0 eq). The resulting polymer was filtered and washed with water, EtOH and ethyl acetate, then dried *in vacuo*. To remove residual Pd, the polymer was stirred in 6 % HCl/7 % H_2O_2 solution for 30 min and Soxhlet extracted with MeOH for 12 h. The polymer was then stirred in 1 M NaOH, washed and dried before use or post-functionalization.

General Procedure for the Post-functionalization of API and APIII

The tolyl polymer, NBS (0.95 eq in respect to tolyl) and AIBN (0.08 eq) were suspended in benzene. The mixture was stirred at 100 °C over night. After cooling, the polymer was filtered, washed with water, EtOH and ethyl acetate, then dried *in vacuo*. Afterwards, the dried polymer was refluxed for 8 h in degassed, dry THF with an excess of *N*,*N*-diisopropyl ethylamine and 3 eq diphenylamine (**API**) or 3,3′-iminobis(*N*,*N*-dimethylpropylamin) (**APIII**). The received macroligand was filtered and dried before use.

SS-NMR-Analysis of Macroligands API-III

API

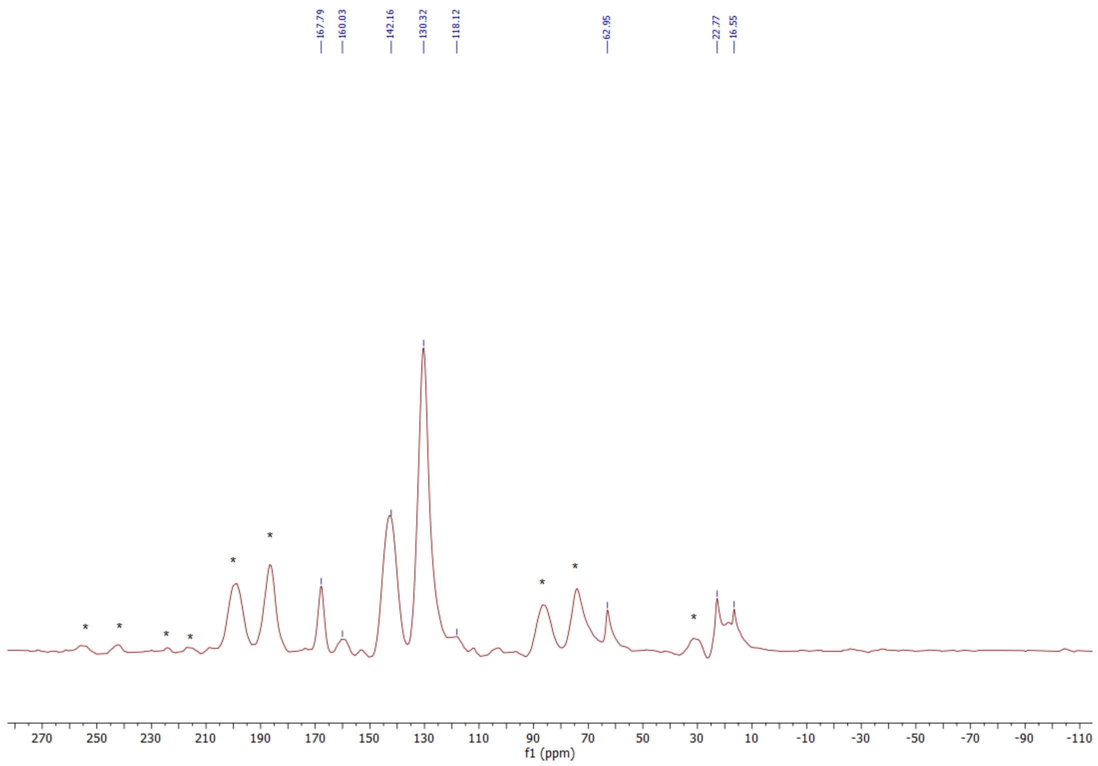

Figure S5: SS-MAS-NMR of macroligand **API** recorded at 7 kHz.

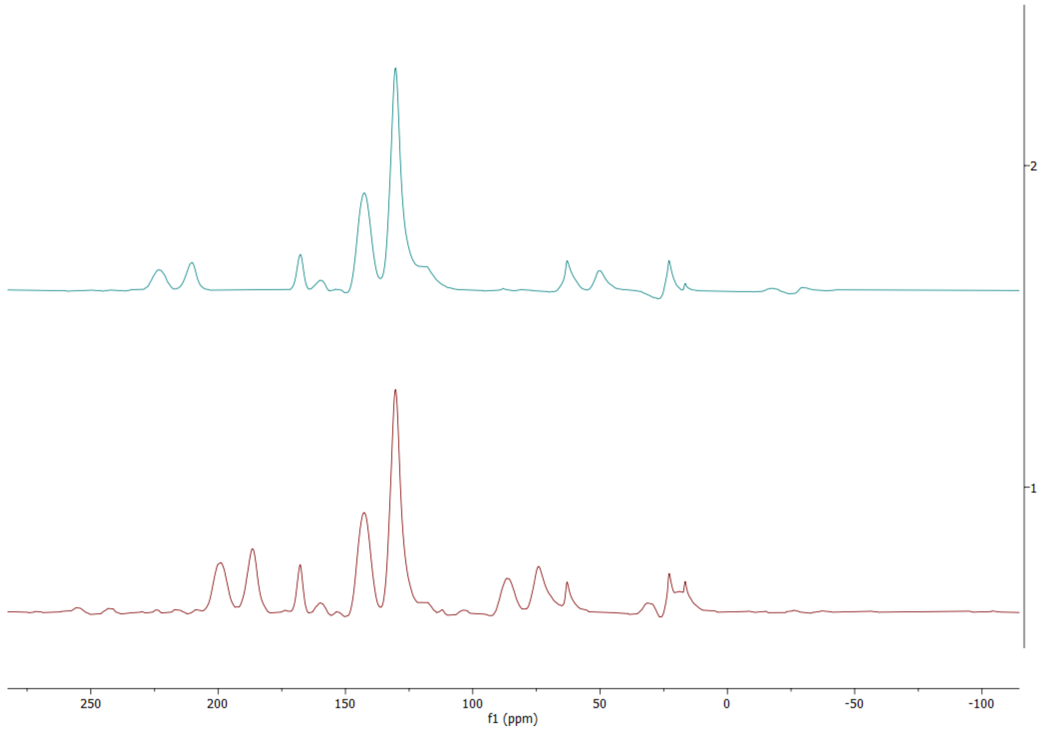

Figure S6: Stacked SS-MAS-NMR of macroligand **API** recorded at 10 (top) and 7 (bottom) kHz.

APII

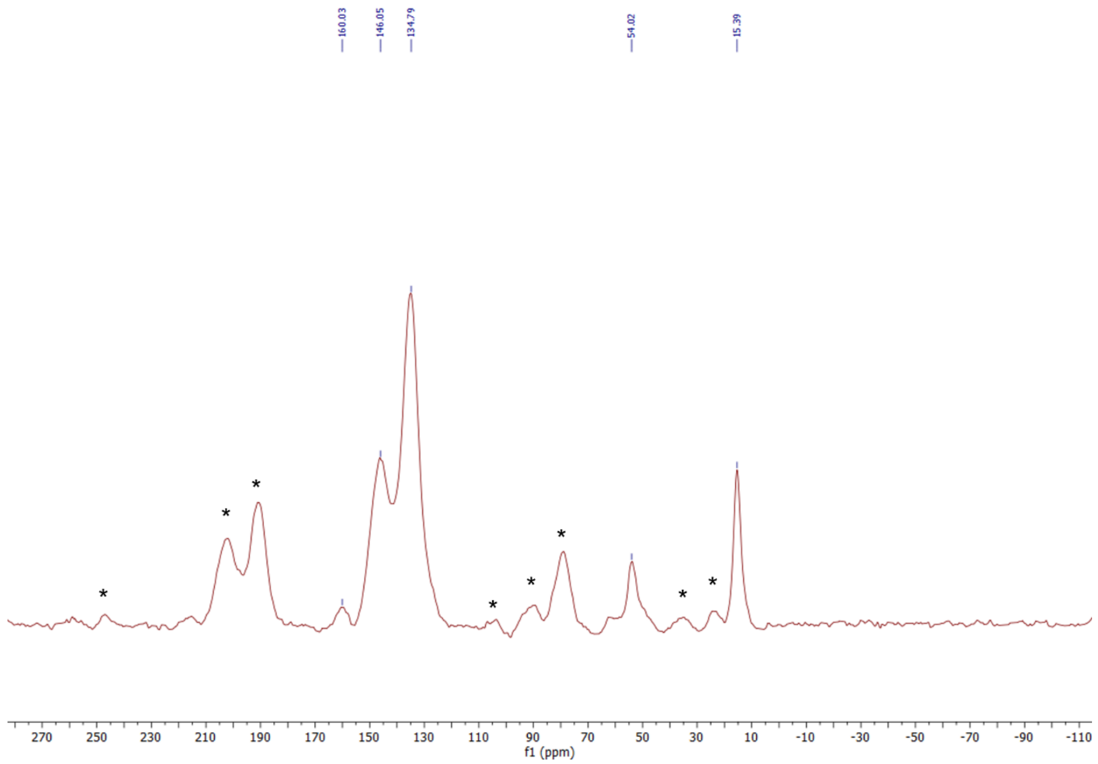

Figure S7: SS-MAS-NMR of macroligand **APII** recorded at 7 kHz.

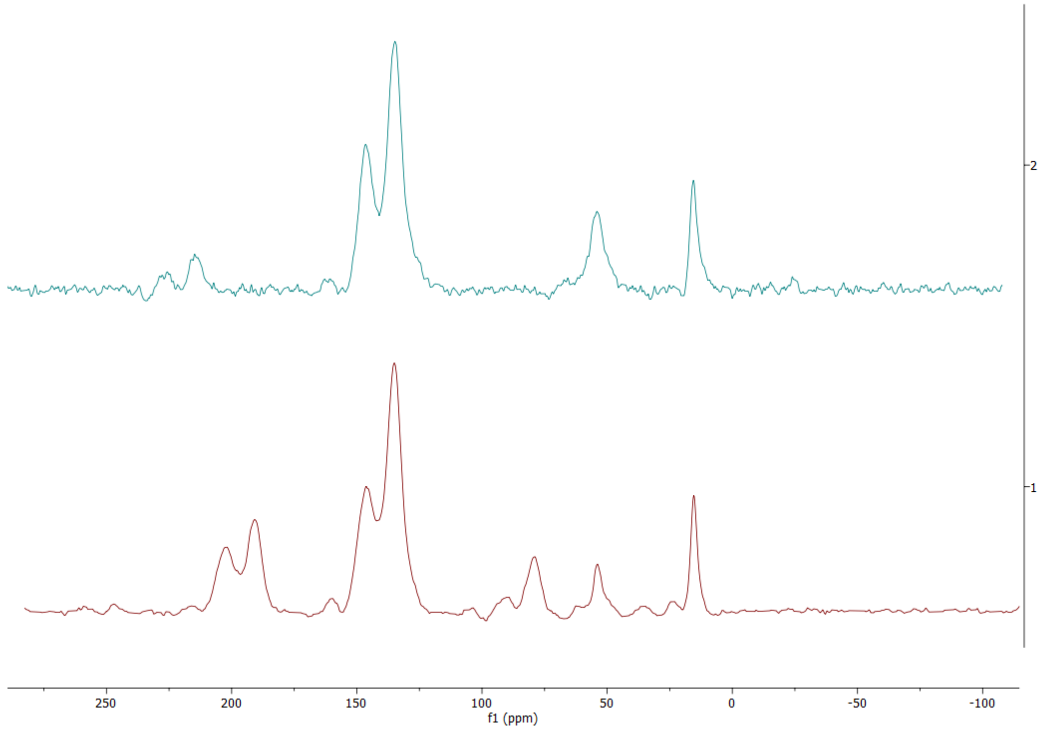

Figure S8: Stacked SS-MAS-NMR of macroligand **APII** recorded at 10 (top) and 7 (bottom) kHz.

APIII

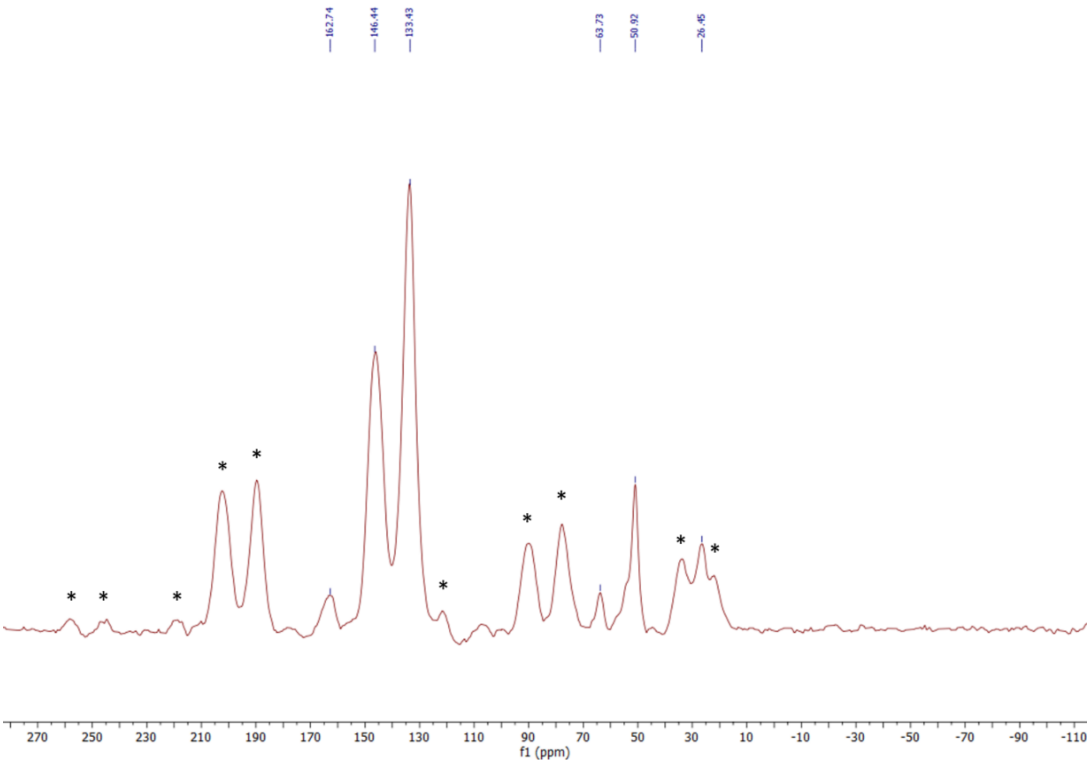

Figure S9: SS-MAS-NMR of macroligand **APIII** recorded at 7 kHz.

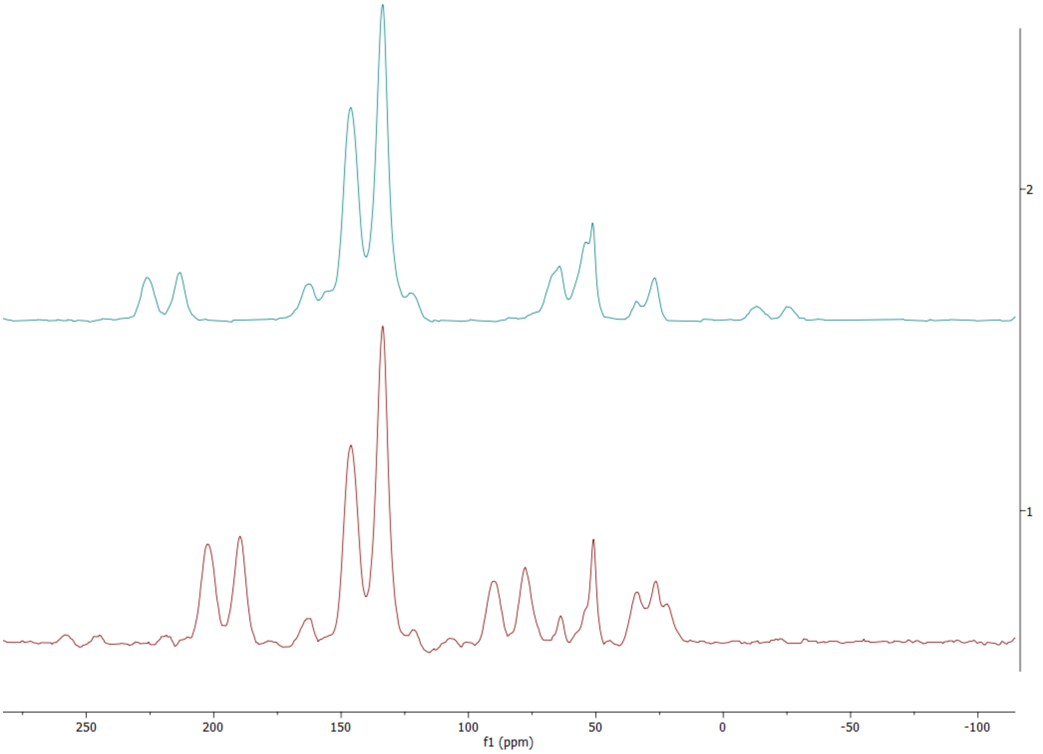

Figure S10: Stacked SS-MAS-NMR of macroligand **APIII** recorded at 10 (top) and 7 (bottom) kHz.

TGA analysis of macroligands

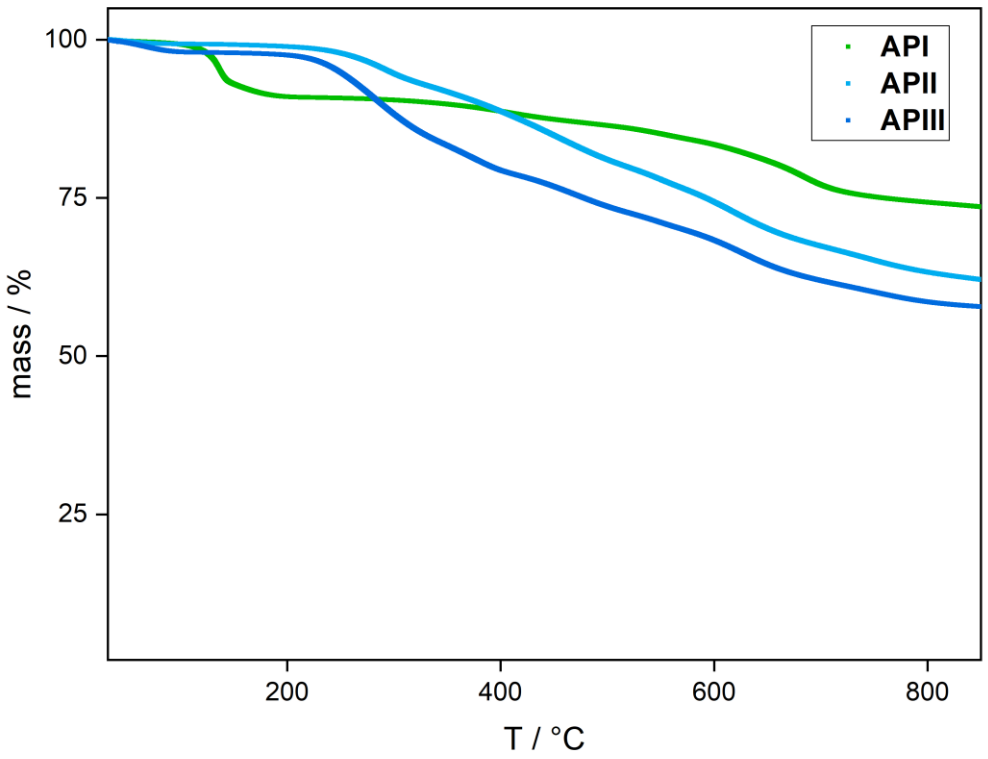

Figure S11: Thermogravimetric analysis of macroligands **API-III**. Conditions: 30-850 °C, 10 °C/min, 150 ml/min N_2 flow.

General Procedure for Reductive Hydroformylation Autoclave Reactions

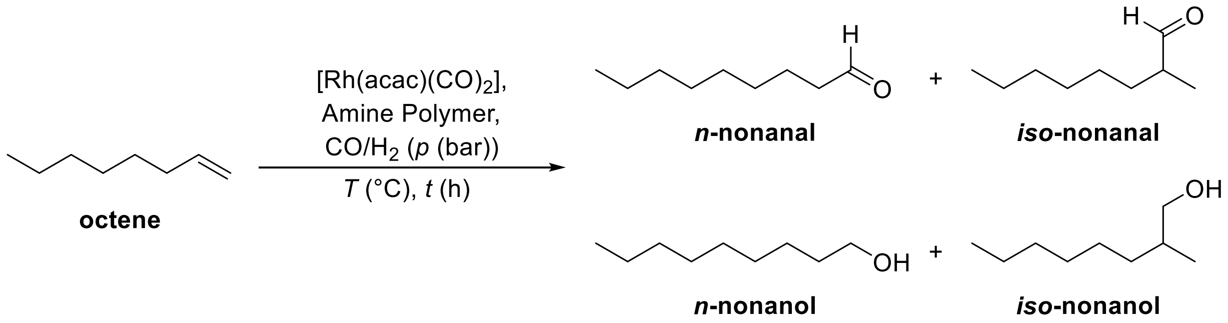

Figure S12: Reductive hydroformylation of octene to *n*-nonanal, *iso*-nonanal, *n*-nonanol and *iso*-nonanol using a rhodium precursor and the **AP**.

Hydroformylation experiments were conducted in 20 mL (total volume) autoclave reactors (Figure S). [Rh(acac)(CO)$_2$], the polymer (**API**: 707 g mol$_N^{-1}$, **APII**: 625 g mol$_N^{-1}$, **APIII**: 247 g mol$_N^{-1}$), the substrate (stock solution with mesitylene as internal standard) and the solvent (if required) were weighed into the glass inlet under normal atmosphere. Most reactions were conducted with a total reaction volume of 2 mL. The inlet was inserted into the autoclave reactor, which was then closed. The autoclave was pressurized with CO (25 bar) once and

depressurized again to remove air before fully pressurizing it to the desired CO/H_2 pressure with CO always being pressurized before H_2. The autoclave was placed in a pre-heated cone and the stirring of 700 rpm was started 10 min afterwards to start the reaction. After the reaction, the reactor was placed in a water bath for cooling, the remaining gas was vented and the autoclave opened. The mixture was filtered through a syringe filter and XRF samples were taken from the filtered product mixture. The filter was then washed with isopropanol (2×1 mL) to remove any product and standard sticking to the polymer. Mesitylene was used as internal standard in all reductive hydroformylation reactions. Samples were analyzed using GC FID and GC MS where necessary.

The autoclave reactors used were fitted with a ball valve or cap (**5**), a needle valve (**4**) through which gases can be applied and released, a rupture disc (**3**) for safety, a manometer (**2**) for pressure recordings and the reactor vessel (**1**) in which the glass inlet was placed to conduct the reaction. The autoclave was placed in a pre-heated aluminum cone for the reaction to have a proper and fast heat distribution.

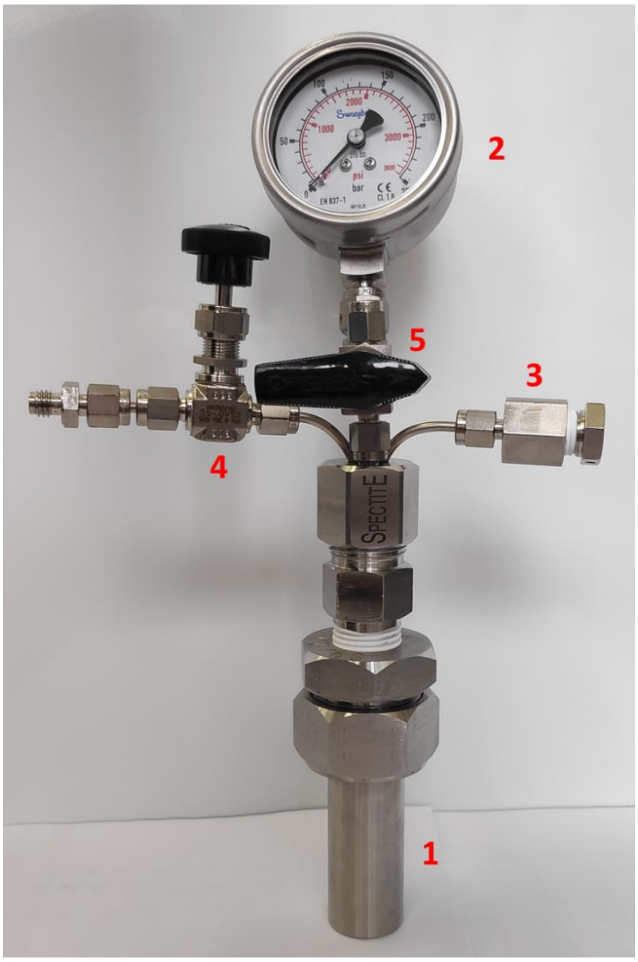

Figure S13: Picture of the applied autoclave consisting of a reaction vessel (**1**), a manometer (**2**), a rupture disk (**3**), a needle valve (**4**) and a ball valve or stopper (**5**).

General Procedure for Reductive Hydroformylation Recycling Experiments

Recycling reactions were carried out as described for the hydroformylation reactions above. After the reaction, the inlet was taken out of the autoclave and placed in a Falcon tube. The product mixture was centrifuged for 5–10 min to settle the polymer particles. The liquid phase was removed using a syringe. XRF samples of the filtered product and diluted GC samples were prepared. After removing most of the liquid, new substrate stock solution was added and the inlet placed back in the autoclave. The reaction was conducted in a next run as described previously.

General Procedure for Hydroformylation Time Profile Experiments

Time profile experiments were conducted in a 300 mL stainless steel reactor supplied by the *Parr Instrument Company* (Figure S). The reactor is fitted with a sampling valve and a fid tube for taking samples and a manometer for reading the pressure. A stock solution of 1-octene with mesitylene as a GC standard were prepared. The rhodium precursor and the polymer were weighed and added as a powder before adding the stock solution. The reactor was flushed with 25 bar of CO once as it was done for the autoclave reactors before pressurizing to the desired reaction conditions. After heating the reactor to the reaction temperature while stirring slowly (ca. 200 rpm), the reaction was started by increasing the stirring speed to 700 rpm. Samples were taken in regular intervals and analysed by GC FID and XRF.

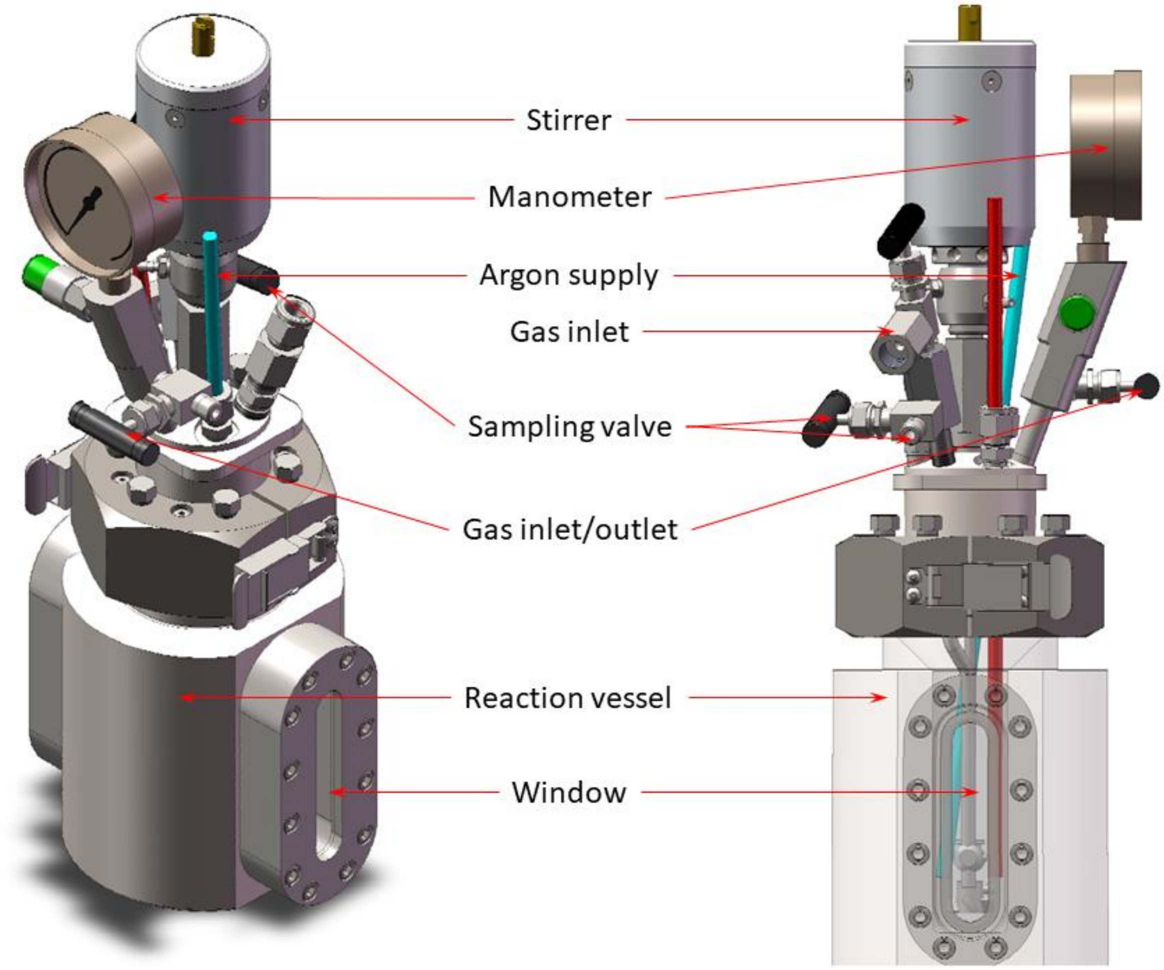

Figure S14: Parr reactor design used for time profile experiments, important features labelled accordingly. Reactor displayed with a 250 mL window fitted vessel but a 300 mL vessel without a window were used for the time profile reactions.

3 GC FID Chromatograms

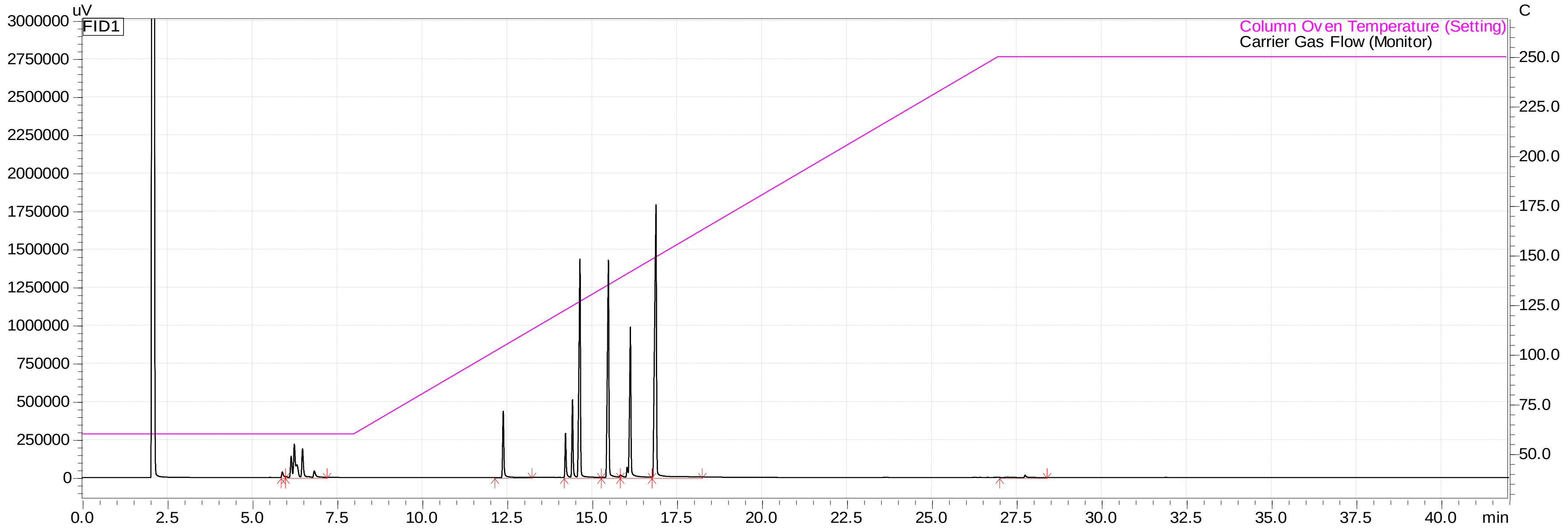

Figure S15: Chromatogram of the reaction mixture after reductive hydroformylation including the temperature profile from 50 °C to 250 °C of the measuring method. Peaks in ascending order: 1-octene, *iso*-octene, octane (same region as *iso*-octenes, identified by GCMS if present), mesitylene, *iso*-nonanal (2-propyl hexanal, 2-ethyl heptanal, 2-methyl octanal), *n*-nonanal, *iso*-nonanol, *n*-nonanol, traces of *n*- and *iso*-nonanoic acid due to the presence of oxygen during the reaction (18–19 min), aldol products (27–28 min).

4 References

[1] J. Husson, L. Guyard, *Heterocycl. Comm.* **2015**, *21*, 199–202.
[2] M. J. Hall, S. O. McDonnell, J. Killoran, D. F. O'Shea, *J. Org. Chem.* **2005**, *70*, 5571–5578.
